# Effects of interactions between variation in dopaminergic genes, traumatic life events, and anomalous self-experiences on psychosis proneness: Results from a cross-sectional study in a nonclinical sample

**DOI:** 10.1192/j.eurpsy.2020.103

**Published:** 2020-11-20

**Authors:** Dorota Frydecka, Kamila Kotowicz, Łukasz Gawęda, Katarzyna Prochwicz, Joanna Kłosowska, Joanna Rymaszewska, Agnieszka Samochowiec, Jerzy Samochowiec, Piotr Podwalski, Edyta Pawlak-Adamska, Elżbieta Szmida, Andrzej Cechnicki, Błażej Misiak

**Affiliations:** 1 Department of Psychiatry, Wroclaw Medical University, 50-367 Wroclaw, Poland; 2 Experimental Psychopathology Lab, Institute of Psychology, Polish Academy of Sciences, Warsaw, Poland; 3 Institute of Psychology, Jagiellonian University, 30-060 Krakow, Poland; 4 Institute of Psychology, Department of Clinical Psychology, University of Szczecin, 71-017 Szczecin, Poland; 5 Department of Psychiatry, Pomeranian Medical University, 71-460 Szczecin, Poland; 6 Department of Experimental Therapy, Laboratory of Immunopathology, Institute of Immunology and Experimental Therapy, Polish Academy of Sciences, 51-114 Wroclaw, Poland; 7 Department of Genetics, Wroclaw Medical University, 50-368 Wroclaw, Poland; 8 Department of Community Psychiatry, Chair of Psychiatry, Medical College Jagiellonian University, Krakow, Poland

**Keywords:** Anomalous self-experiences, childhood trauma, psychosis, schizophrenia, stress

## Abstract

**Background:**

There is a growing number of studies showing interactions between genetic polymorphisms associated with dopaminergic neurotransmission and traumatic life events (TLEs) on a risk of psychotic-like experiences (PLEs). Anomalous self-experiences (ASEs) have been associated both with TLEs as well as with PLEs. However, it remains unknown what is the role of ASEs in the complexity of gene–environment interactions on the emergence of PLEs.

**Patients and methods:**

We included 445 young adults—university students from three big cities in Poland. We used the Traumatic Events Checklist to assess TLEs, the Inventory of Psychotic-Like anomalous self-experiences in order to measure ASEs, and the Prodromal Questionnaire (PQ16) to record the level of PLEs. The following gene polymorphisms, related to dopaminergic neurotransmission, were determined: the catechol-*O*-methyltransferase (*COMT*) rs4680 polymorphism, the dopamine D2 receptor (*DRD2*) rs6277 polymorphism, and the dopamine transporter 1 (*DAT1*) rs28363170 polymorphism.

**Results:**

There was a significant effect of the interaction between the *DAT1* polymorphism, a severity of ASEs, and a history of TLEs on the level of PLEs. Among the *DAT1* 10R/10R homozygotes with low level of ASEs, a severity of PLEs was significantly higher in individuals with a history of any TLEs. Higher scores of the PQ16 were associated with a greater severity of ASEs both in the *DAT1* 9R allele carriers and the *DAT1* 10R/10R homozygotes.

**Conclusion:**

Our findings imply that genetic liability related to aberrant dopamine transport might impact the association between TLEs and PLEs in subjects with high levels of ASEs.

## Introduction

Anomalous self-experiences (ASEs) are among the first symptoms that appear in the prodrome, predicting the development of psychosis, and are common in people with schizophrenia [1]. ASEs have a long history in psychopathology research dating back to Bleuler, who claimed that schizophrenia is primarily a disorder of the self [2]. Current body of research suggests that disordered selfhood may be a central phenotypic feature of schizophrenia [2,3]. Self-disorders present in different forms at varying magnitudes in the premorbid, prodromal, first episode, chronic, and recovery phases of the disorder [4]. Moreover, they hyperaggregate not only in schizophrenia but also in its spectrum diagnoses, such as schizotypal disorder, nonorganic, and nonaffective psychoses, as well as among first-degree relatives of schizophrenia patients [5]. Interestingly, ASEs have been shown to be moderately to highly stable over time [6,7].

ASEs are disturbances in the subjective experience of the self. There are various types of ASEs, such as disturbed sense of presence (basic self-awareness), anomalous bodily experiences (perceived morphological change), anomalous cognitive processes (perceptualization of inner speech), self-demarcation (confusion between oneself and other people), and existential reorientation (self-reference), all of which are interrelated [8]. It has been postulated that self-disturbances are manifested as diminished self-affection and hyperreflexivity in patients with schizophrenia [3]. Diminished self-affection refers to a disturbance in self-agency or a diminished sense of possession of one’s experiences or disturbance in the perception that one is acting out one’s behavior. Hyperreflexivity refers to excessive attention to self-processes that are normally experienced unconsciously [4].

ASEs have been shown to positively correlate with psychotic-like experiences (PLEs) and self-consciousness while negatively correlate with self-report and self-concept clarity as well as self-esteem [1]. Higher scores are obtained in people with positive schizotypy compared to negative schizotypy as well as in patients with schizophrenia compared with healthy controls [1]. A prospective study on nonclinical help-seeking adolescents has shown that ASEs can serve as a clinical marker of risk for schizophrenia-spectrum disorder [7]. Another study on nonpsychotic young adults has shown that a combination of high level of PLEs and self-disturbances may capture the highest risk of psychosis in the general population associated with cognitive biases characteristic for psychosis [9]. In a clinical sample, ASEs have been shown to predict the onset of schizophrenia-spectrum disorders within 5 years after hospitalization due to a nonpsychotic disorder [10]. Moreover, a relationship between ASEs and the presence of attenuated positive symptoms, as well as with cognitive or cognitive-perceptive basic symptoms, was found in individuals at clinical high risk (CHR) for psychosis [11]. Finally, ASEs were also associated with psychosocial functioning [12], social functioning [13], and global functioning [11,14] in CHR samples.

The dopamine hypothesis is the longest standing theory of schizophrenia etiology, and increased striatal dopaminergic activity is the most frequently replicated pathophysiological correlate of psychotic symptoms [15,16]. Studies investigating the way risk factors of psychosis affect the dopamine system, coupled with longitudinal studies during the prodrome, allow for a more refined understanding of what leads to the onset of psychotic disorders. Various studies have suggested that dopaminergic abnormalities exist in CHR individuals and include elevated peripheral dopamine metabolites [17] or differences in dopaminergic receptor densities [18]. It has been shown that dopamine synthesis capacity is raised in at-risk individuals [19,20], and it is positively associated with the severity of prodromal symptoms [21]. Moreover, antipsychotic treatment trials have demonstrated efficacy of dopamine blockage in reducing prodromal symptoms severity [22,23]. Numerous studies have pointed that aberrant dopamine release might be associated with expression of genes-encoding proteins involved in dopamine synthesis, metabolism, neurotransmission, and signaling [24].

Meta-analytic studies point to the association between traumatic life events (TLEs) and the risk of psychosis [25,26] and transition to psychosis among CHR individuals [27]. Stress has also been identified as a marker for the occurrence of subclinical psychosis in a large-scale female twin study [28]. Moreover, it has been shown that TLEs have an impact on dopaminergic neurotransmission. Greater striatal dopamine release in response to social stress has been observed both in individuals at risk of psychosis [29,30] and those with schizophrenia [31]. Interestingly, greater dopamine synthesis capacity [32] and release [33] have been found among adult individuals exposed to childhood adversity.

“Gene–environment studies have demonstrated interactions between genetic and psychosocial risk factors for psychosis [34]. There are several studies showing how genes associated with dopaminergic neurotransmission mediate the relationship between TLEs, dopamine, and psychosis (for review, see [35]). There are also studies showing the relationship between dopamine transmission and ASEs as well between ASEs and PLEs. Moreover, the impact of TLEs on disruptions of sense of self and agency has been discussed previously [36]. However, there are no studies looking at the between TLEs, dopamine, and psychosis proneness while taking into account the severity of ASEs. Therefore, in this study, we aimed to test the effect of interaction between variation in genes playing role in dopaminergic neurotransmission (the *COMT* rs4680, the *DRD2* rs6277, and the *DAT1* rs28363170 variable number tandem repeat (VNTR) and TLEs on a risk of PLEs, taking into account the impact of ASEs, in a nonclinical sample.”

## Methods

### Participants

A detailed description of methodological issues related to our study was published in our previous article [37]. We included 535 undergraduate and postgraduate university students from three big cities in Poland (Wroclaw, Krakow, and Szczecin). A detailed description of sampling procedures was provided in our previous articles [9,37,38]. All participants were of Caucasian origin. The study was approved by the Ethics Committee at Wroclaw Medical University. All participants gave written informed consent.

### Measures

#### The Traumatic Events Checklist

We used the Traumatic Events Checklist to assess TLEs [39]. It is a self-report questionnaire including 29 items. It captures six types of traumatic events. Emotional abuse is related to being belittled, teased, called names, threatened verbally, or unjustly punished by parents, brothers, and sisters. Emotional neglect is related to being left alone or insufficient affection given by parents. Physical abuse is related to being hit, tortured, or wounded by parents, brothers, or sisters. Threat to life refers to situations of threat to life from another person, such as during a crime. Sexual harassment refers to acts of a sexual nature that do not involve physical contact by parents, brothers, or sisters. Sexual abuse is defined as unwanted sexual acts involving physical contact by parents, brothers, or sisters.

#### The Inventory of Psychotic-Like Anomalous Self-Experiences

We used the Inventory of Psychotic-Like Anomalous Self-Experiences (IPASE) in order to measure self-disorders. The IPASE is a brief self-report alternative to phenomenological interviews that can be used in the general population, at-risk, and clinical samples to assess minimal self-disturbances [1]. It has been developed based on a comprehensive phenomenological interview for self-disorders—the Examination of Anomalous Self Experience [40]. The IPASE consists of 57 items that can be grouped into five factors [1]. The factor cognition consists of items related to difficulties with thought processes. The second factor, self-awareness, and presence contain items related to loss of basic self or identity and a loss of a connection to the world. The third factor, consciousness, includes items about disturbances in intentionality and difficulty with distinguishing between imagination and reality. The fourth factor, somatization, includes items involving disturbances in bodily experiences, such as feeling like the body was changing shape or difficult to control, as well as thought of not feeling present either psychically or physically within one’s own body. The fifth factor, demarcation/transitivism, consists of items related to a disintegrating boundary between the self and the world or a feeling of nonexistence [1]. The IPASE provides a shorter and more easily administered alternative to phenomenological interviews [1]. The IPASE scores are reliable and valid in a general sample of people with high levels of schizotypy and at risk for the future development of psychosis as well as in individuals with schizophrenia [1].

#### The Prodromal Questionnaire 16

We used the 16-item Prodromal Questionnaire (PQ16) to screen for PLEs [41]. It is a 16-item self-report questionnaire that consists of nine items of the perceptual abnormalities/hallucinations subscale, five items referring to unusual thought content/delusional ideas/paranoia, and two negative symptoms. The perceptual aberrations are overrepresented since perceptual aberrations are primary psychotic phenomena, and that delusions are explanations of these abnormal sensory experiences. Perceptual aberrations are seen as a first phenomenon leading to psychosis. We excluded items one and seven from data analysis because they do not capture positive PLEs. Our analysis was limited to a number of various PLEs experienced by participants.

### Genotyping

DNA samples were obtained via buccal swabs. The single nucleotide polymorphisms: *COMT* rs4680 (Val158Met) and the *DRD2* rs6277 (957C>T) were genotyped with the allelic discrimination technique with the use of validated and predesigned TaqMan®SNP Genotyping Assays (C__25746809_50 and C__11339240_10) according to the manufacturer’s instructions (ThermoFisher Scientific, Inc., USA). *DAT1* VNTR rs28363170 polymorphism was genotyped through PCR with pair of primer, where the forward one was labeled with 6-FAM followed by capillary electrophoresis in the presence of the GeneScan™ 600 LIZ® Size Standard on 3500 Genetic Analyzer (Applied Biosystems). Individual genotypes were called according to peak size on GeneMapper® Software version 4.0., being the 9-allele (440 bp) and the 10-allele (480 bp).

### Statistics

Agreement of genotypes distribution with the Hardy–Weinberg equilibrium (HWE) was assessed by comparing observed and expected distributions using the *χ*^2^ test. Bivariate comparisons were performed using the Mann–Whitney *U* test. The Spearman rank correlation coefficients were used to assess correlations. Multiple linear regression was performed to test the effects of variation in dopaminergic genes and a history of any TLEs on the number of PLEs. Main effects were represented by a history of any TLEs, the allele status (*COMT*: the Met allele carriers vs. the Val/Val homozygotes; *DRD2*: the C allele carriers vs. the TT homozygotes; *DAT1*: the 9R allele carriers vs. the 10R/10R homozygotes), and the level of ASEs. The level of ASEs was dichotomized to high level (individuals scored above the mean score of the IPASE) and low level (individuals scored below the mean score of the IPASE). Age and sex were added as covariates. Indeed, the onset of psychosis usually appears in the adolescence or early adulthood [42]. In turn, sex differences might impact various clinical characteristics of individuals at clinical high risk of psychosis [43]. Firstly, we tested the effects of covariates (the first block of independent variables, model 1). Next, the main effects were implemented (the second block of independent variables, model 2). The third block of independent variables (model 3) included the two-way interactions (allele status × TLEs, allele status × ASEs, and TLEs × ASEs). Finally, we inputted the three-way interaction term (allele status × TLEs × ASEs, model 4). Posthoc analyses were based on bivariate comparisons using the Mann–Whitney U test. Results were considered significant if the *p*-value was less than 0.05. Statistical analysis was performed using the Statistical Package for Social Sciences, version 20 (SPSS, Inc., Chicago, Illinois, USA).

## Results

General characteristics of participants were presented in Table 1. Distribution of the *COMT* rs4680 and the *DAT1* VNTR 9R/10R genotypes was in agreement with the HWE (*COMT*: *χ*^2^ = 0.07, *p* = 0.791; *DAT1* 9R/10R VNTR: *χ*^2^ = 1.78, *p* = 0.183). However, the distribution of the *DRD2* genotypes did not follow the HWE (*χ*^2^ = 6.31, *p* = 0.012). As expected, a history of TLEs was associated with significantly higher scores of the IPASE (Table 2). There were significant positive correlations between the IPASE scores and the PQ16 score (Table 3).

**Table 1.** General characteristics of the sample.

	Mean ± SD or *n* (%)
Age, years	23.4 ± 3.0
Sex (M/F)	129 (29.0)/316 (71.0)
Clinical diagnosis	38 (8.5)
TEC (positive history of any TLEs)	253 (56.8)
PQ-16 (total score)	5.4 ± 5.4
PQ-16 (without items 1 and 7)	4.2 ± 4.6
ASEs (low level/high level)	169 (38.0)/276 (62.0)
*COMT* rs4680	
Val/Val	105 (23.8)
Val/Met	218 (49.3)
Met/Met	119 (26.9)
*DRD2* rs6277	
CC	124 (27.9)
CT	196 (44.0)
TT	125 (28.1)
*DAT1* rs28363170	
9R/9R	28 (8.6)
9R/10R	118 (36.3)
10R/10R	179 (55.1)

Abbreviations: ASE, anomalous self-experience; COMT, catechol-*O*-methyltransferase; DAT1, dopamine transporter 1; DRD2, dopamine D2 receptor; PQ-16, Prodromal Questionnaire 16; TEC, Traumatic Events Checklist; TLEs, traumatic life events; SD, standard deviation.

**Table 2.** The IPASE scores with respect to a history of TLEs.

IPASE	TLEs (+)	TLEs (−)
IPASE—cognition	9.8 ± 4.1	8.5 ± 2.7
IPASE—self-awareness	36.4 ± 14.8	30.6 ± 10.0
IPASE—consciousness	13.7 ± 5.0	11.0 ± 4.4
IPASE—somatic	28.1 ± 10.4	24.1 ± 8.0
IPASE—demarcation/transitivism	7.9 ± 3.0	6.8 ± 2.3
IPASE—total score	95.9 ± 33.6	80.9 ± 24.1
PQ16 score	4.9 ± 5.1	3.1 ± 3.7

Abbreviations: IPASE, inventory of psychotic-like anomalous self-experiences; PQ-16, Prodromal Questionnaire 16; TLEs, traumatic life events.

Data expressed as mean ± SD.

*p* < 0.001 for all comparisons.

**Table 3.** Correlations between the IPASE scores and the PQ16 score.

The IPASE score	PQ16
Cognition	*r* = 0.479
Self-awareness	*r* = 0.515
Consciousness	*r* = 0.549
Somatic	*r* = 0.550
Demarcation/transitivism	*r* = 0.430
Total score	*r* = 0.590

Abbreviations: IPASE, inventory of psychotic-like anomalous self-experiences; PQ-16, Prodromal Questionnaire 16.

Spearman’s rank correlation coefficients.

*p* < 0.001 for all correlations.

Results of multiple linear regression were presented in Table 4. There were significant main effects of age and a severity of ASEs on the PQ16 scores in all models. The effect of a history of TLEs on the PQ16 score was significant only in the model 2 for all tested gene polymorphisms. No significant main effects of gene polymorphisms on the PQ16 scores were found. However, there was a significant effect of the interaction between the *DAT1* 9R/10R VNTR and a severity of ASEs on the PQ16 score (model 4). Effects of the interaction between a history of TLEs and the level of ASEs on the PQ16 score were significant in the models 3 and 4 for the *COMT* rs4680 and the *DRD2* rs6277 polymorphisms. Similarly, the effects of a three-way interaction (the *DAT1* 9R/10R allele status × TLEs × ASEs) on the PQ16 score were also significant. The model 4 that included the *DAT1* 9R/10R allele status explained the largest proportion of variance in the PQ16 score (*R*^2^ = 0.355).

**Table 4.** Results of multiple linear regression analysis.

Model	Effect	*DAT1*				*COMT*				*DRD2*		
		*R*^2^/Δ*R*^2^	B	SE	*p*	*R*^2^/Δ*R*^2^	B	SE	*p*	*R*^2^/Δ*R*^2^	B	SE
1	Age	0.103/0.103*	−0.449	0.083	**<0.001**	0.110/0.110*	−0.454	0.068	**<0.001**	0.115/0.115*	−0.475	0.070
	Sex		−0.693	0.549	0.207		−0.728	0.452	0.108		−0.839	0.466
2	Age	0.324/0.221*	−0.347	0.073	**<0.001**	0.311/0.201*	−0.335	0.061	**<0.001**	0.314/0.199*	−0.350	0.063
	Sex		−0.672	0.479	0.162		−0.651	0.399	0.104		−0.722	0.412
	ASEs		4.349	0.458	**<0.001**		3.776	0.381	**<0.001**		3.838	0.390
	TLEs		0.958	0.447	**0.033**		1.168	0.361	**0.001**		1.275	0.372
	Allele		−0.239	0.436	0.583		−0.336	0.416	0.420		0.747	0.406
3	Age	0.337/0.013	−0.342	0.073	**<0.001**	0.330/0.018*	−0.348	0.061	**<0.001**	0.332/0.018*	−0.359	0.063
	Sex		−0.654	0.477	0.171		−0.586	0.397	0.140		−0.663	0.409
	ASEs		3.009	0.866	**0.001**		2.639	0.638	**<0.001**		2.422	0.673
	TLEs		−0.376	0.729	0.607		1.046	0.494	**0.035**		0.187	0.503
	Allele		−1.338	0.743	0.073		0.541	0.628	0.389		0.373	0.670
	ASEs × TLEs		1.625	0.959	0.091		1.749	0.764	**0.023**		2.412	0.779
	Allele × ASEs		0.366	0.924	0.693		0.313	0.922	0.734		−0.204	0.826
	Allele × TLEs		1.443	0.900	0.110		−1.936	0.845	**0.022**		0.869	0.819
4	Age	0.355/0.018*	−0.344	0.072	**<0.001**	0.335/0.006	−0.344	0.061	**<0.001**	0.332/0	−0.359	0.063
	Sex		−0.711	0.472	0.133		−0.611	0.396	0.123		−0.663	0.410
	ASEs		4.584	1.011	**<0.001**		2.195	0.678	**0.001**		2.468	0.749
	TLEs		0.601	0.794	0.450		0.783	0.512	0.127		0.211	0.530
	Allele		−0.317	0.813	0.697		0.090	0.670	0.893		0.420	0.748
	ASEs × TLEs		−1.178	1.346	0.382		2.471	0.851	**0.004**		2.338	0.937
	Allele × ASEs		−3.145	1.507	**0.038**		2.562	1.499	0.088		−0.347	1.297
	Allele × TLEs		−0.373	1.084	0.731		−0.955	0.988	0.334		0.777	1.042
	Allele × TLEs × ASEs		5.540	1.892	**0.004**		−3.598	1.894	0.058		0.240	1.684

Abbreviations: ASEs, anomalous self-experiences; COMT, catechol-*O*-methyltransferase; DAT1, dopamine transporter 1; TLEs, traumatic life events; DRD2, dopamine D2 receptor.

Significant effects (*p* < 0.05) were marked with bold characters.

* Significant *R*^2^ changes (*p* < 0.05).

**Figure 1.**
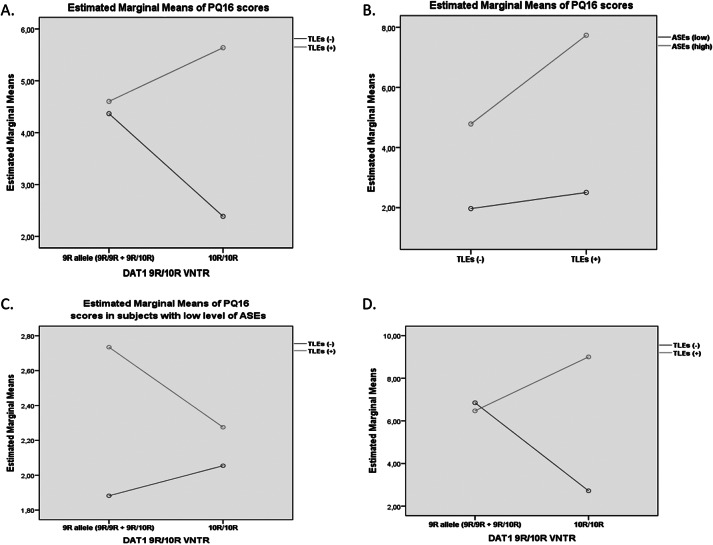
Effects of the interactions between the dopamine transporter 1 (*DAT1*) 9R/10R polymorphism, a history of traumatic life events (TLEs), and a severity of anomalous self-experiences (ASEs) on the score of the Prodromal Questionnaire 16 (PQ16). (A) Effects of the interaction between the *DAT1* 9R/10R polymorphism and the level of ASEs on the PQ16 score. (B) Effects of the interaction between the *DAT1* 9R/10R polymorphism and a history of TLEs on the PQ16 score in individuals with low level of ASEs. (C) Effects of the interaction between the *DAT1* 9R/10R polymorphism and a history of TLEs on the PQ16 score in individuals with high level of ASEs. Error bars represent 95% confidence interval (CI).

Results of posthoc analyses were shown in Figure 1 and Supplementary Material. Higher scores of the PQ16 were associated with a greater severity of ASEs both in the *DAT1* 9R allele carriers and the *DAT1* 10R/10R homozygotes (*p* < 0.001,Figure 1 and Supplementary Table 1). In participants with a high level of ASEs, a history of TLEs was associated with significantly higher scores of PQ16 in the *DAT1* 10R/10R homozygotes (8.36 ± 6.06 vs. 4.47 ± 3.34, *p* = 0.015) but not in the *DAT1* 9R allele carriers (Figure 1 and Supplementary Table 2). These results were not significant in the group with low levels of ASEs.

Model 4 for DAT1 explained the largest proportion of variance in the PQ16 score measured by the *R*^2^ statistic (Table 4). Adding a three-way interaction term significantly increased the *R*^2^ statistic. In turn, adding a three-way interaction term to the models with the DRD2 and COMT polymorphisms did not change the *R*^2^ statistic significantly.

## Discussion

In our previous articles, we have shown that a path from trauma to psychosis proneness leads via cognitive biases and self-disorders [38] and that the combination of high level of PLEs together with self-disturbances captures the highest risk of psychosis in the general population [9]. Our main finding from genetic studies so far was that among participants with high levels of cognitive biases, there is an interaction between *COMT* gene polymorphism and a history of TLEs on the severity of PLEs [37]. In our current study, we wanted to see whether not only the level of cognitive biases but also the level of self-disorders influence the interaction between genetic polymorphisms in the genes influencing dopaminergic neurotransmission (*COMT*, *DRD2*, and *DAT1*) and a history of TLEs on the severity of PLEs.

Findings from our study imply that TLEs might increase severity of PLEs only in 10R/10R homozygotes of the *DAT1* 9R/10R VNTR polymorphism. *DAT1* is a protein regulating dopamine synaptic levels by limiting dopamine receptor activation and facilitating neuronal dopamine reuptake. Several *DAT1* gene polymorphisms have been described, among these, there is a 40 bp VNTR that most commonly occurs as 9 or 10 tandem repeats (R). There is evidence that this polymorphism is functional, altering gene expression. According to current models, reduced *DAT1* expression should amplify phasic DA signals [44]. It has been found that healthy individuals with the *DAT1* 10R/10R genotype show lower striatal transporter binding [45], while the *DAT1* 9R allele carriers present with increased striatal dopamine transporter availability [46,47]. A recent meta-analysis confirmed that the *DAT1* 9R/10R VNTR polymorphism regulates dopamine activity in the striatal brain regions independent of the presence of neuropsychiatric illnesses [48]. Thus, it can be hypothesized that additionally to increased striatal dopamine activity associated with the *DAT1* gene polymorphism, exposure to TLEs further increases dopaminergic neurotransmission, leading to increased risk of PLEs. This is in line with schizophrenia risk model which requires two hits in order to generate the clinical phenotype. The first hit is associated with early priming in a genetically predisposed individuals, while the second hit is usually an environmental insult occurring at certain neurodevelopmental stages [49]. Although neither population-based association study [50] nor family-based association study [51] showed the significance of the *DAT1* 9R/10R VNTR polymorphism in the development of schizophrenia, these studies did not control for the effects of TLEs. Partially, the interaction between TLEs and the *DAT1* polymorphism could be due to the fact that genetic variants in dopaminergic genes are associated with considerable interindividual variation in the ability to acquire and extinguish conditioned fear reactions [52]. In particular, it has been shown that higher learning rates during fear extinction are observed in the *DAT1* 9R-carriers as compared to noncarriers [53].

There are few studies so far showing that the *DAT1* polymorphisms are associated with negative experiences in the childhood and negative parenting. A study on male and female twins showed that men with the 9R/9R genotype report less sexual abuse experiences in comparison to other genotypes, while in women, there was an association between the 9R/9R genotype with less experiences of emotional abuse in comparison to other genotypes [54]. These results suggest that either some experiences of childhood abuse may be genetically influenced, or children carrying the specific genotype have temperamental traits that make them more vulnerable to abuse. Another scenario is that children inherit from their parent the genotype that is associated with certain behavior (e.g., antisocial behavior). On the other hand, the association between maternal *DAT1* 9R/10R genotype and negative parenting has been found and appeared to be significantly stronger among mothers whose children were highly disruptive and noncompliant during the mother–child interactions, suggesting gene–environment interaction [55].

There are also mixed findings from studies showing an interaction between negative experiences and psychiatric symptomatology in nonpsychotic disorders. There is evidence that an interaction between the *DAT1* VNTR polymorphism and TLEs has an impact on symptomatology in attention deficit hyperactivity disorder (ADHD) patients. The authors showed that adolescents being homozygous for the 10R of the *DAT1* VNTR polymorphism who grew up in greater psychosocial adversity exhibit significantly more ADHD symptoms in comparison to adolescents with other genotypes or who lived in less adverse family environments [56]. On the other hand, it has been shown that children with 10R/10R genotype have lower level of conduct problems under the condition of low level of positive maternal emotions than those patients with the 9R/9R and 10R/9R genotypes [57]. The *DAT1* gene 9R variant has been associated with child negative affect expressed toward the parent during parent–child interactions, and parents of children with the 9R allele have been found to exhibit more hostility and lower guidance/engagement than parents of children without the 9R allele [58]. Moreover, the *DAT1* single nucleotide polymorphism (rs40184) has been shown to moderate the effect of perceived maternal rejection on the onset of major depressive disorder and suicidal ideation in male adolescents [59].

Our further analysis revealed that the interaction between TLEs and the *DAT1* polymorphism appears only in individuals with high levels of ASEs. Although there are no studies so far that directly associate dopaminergic neurotransmission with ASEs, it has been shown that self-awareness is regulated by dopamine [60,61] via the GABA system through the medial prefrontal cortex and anterior cingulate cortex [62] (for review, see [63]). The current model of ASEs is based on a combination of empirical findings, clinical experience, and phenomenological considerations; however, there is a growing body of research trying to link phenomenology with neurobiology despite technical and conceptual challenges (for review, see [64]). A meta-analytic study showed that the cortical midline system may be responsible for the self-referential processing—basic sense of self—and changes in this region are related to psychotic symptoms as well as they are among the earliest alterations observed in schizophrenia [65]. Aberrant dopamine transmission is thought to disturb the salience of external and internal stimuli, and self-disturbances and psychotic symptoms may arise as patients experience these abnormally salient stimuli [66]. On the other hand, aberrant salience may result in diminished self-affection and hyperreflexivity, contributing to disturbed basic selfhood [64].

Various limitations of this study need to be taken into consideration. Firstly, our sample was not large, and replication of findings in an independent sample was not performed. Secondly, a cross-sectional study design does not provide insights into causal associations. Finally, the use of a nonclinical sample can also be perceived as potential limitation and does not allow for generalization of findings over patients with psychotic disorders. However, this approach is characterized by a lack of confounding factors related to clinical symptoms, cognitive impairments, comorbidities, and medication effects. Finally, the use of self-reports has been raised as a limitation. However, there is evidence that assessment with self-reports is characterized by high validity and reporting stability.

In summary, our results imply that the effect of interaction between the *DAT1* VNTR polymorphism and TLEs on the level of PLEs is limited to individuals with high levels of ASEs. These results provide novel insights into the complexity of gene– environment interactions by showing that simple models might be insufficient in addressing the complexity of how genetic predisposition together with environmental stress might lead to subclinical psychotic experiences. Given that assessment of ASEs provides a unified approach to understand highly diverse symptomatology of schizophrenia, longitudinal and neurobiological studies investigating their basis can unravel new pathways of psychosis development.

## Data Availability

The data that support the findings will be available in upon request from the corresponding author.

## Supplementary material

For supplementary material accompanying this paper visit http://dx.doi.org/10.1192/j.eurpsy.2020.103. click here to view supplementary material
