# Supplementary material for: Effects of interactions between variation in dopaminergic genes, traumatic life events, and anomalous self-experiences on psychosis proneness: Results from a cross-sectional study in a nonclinical sample
Source: Eur Psychiatry. 2020 Nov 20;63(1):e104. doi: 10.1192/j.eurpsy.2020.103 (PMC8057383; doi:10.1192/j.eurpsy.2020.103)
Supplement: Supplementary file 1 [file S0924933820001030sup001.docx]

**Supplementary**

Supplementary Table 1. The PQ16 score with respect to the *DAT1* gene polymorphism and the level of ASEs.

|  | *DAT1* 9R allele  (9R/9R, 9R/10R) | *DAT1* 10R/10R genotype | p |
| --- | --- | --- | --- |
| Low level of ASEs | 2.57 ± 2.75, n = 94 | 2.00 ± 2.31, n = 106 | 0.127 |
| High level of ASEs | 6.71 ± 5.66, n = 49 | 7.41 ± 5.75, n = 70 | 0.453 |
| p | **< 0.001** | **< 0.001** | - |

Abbreviations: ASEs – anomalous self-experiences, DAT1 – dopamine transporter

Supplementary Table 2. The PQ16 score with respect to the level of ASEs, a history of TLEs and the DAT1 gene polymorphism.

|  | Low level of ASEs | | p | High level of ASEs | | p |
| --- | --- | --- | --- | --- | --- | --- |
|  | *DAT1* 9R allele  (9R/9R, 9R/10R) | *DAT1* 10R/10R genotype |  | *DAT1* 9R allele  (9R/9R, 9R/10R) | *DAT1* 10R/10R genotype |  |
| TLEs(+) | 2.69 ± 2.46, n = 55 | 2.16 ± 2.14, n = 57 | 0.265 | 6.26 ± 5.84, n = 27 | 8.36 ± 6.06, n = 53 | 0.082 |
| TLEs(-) | 2.41 ± 3.14, n = 39 | 1.84 ± 2.52, n = 49 | 0.314 | 7.27 ± 5.50, n = 22 | 4.47 ± 3.34, n = 17 | 0.181 |
| p | 0.240 | 0.163 | - | 0.657 | **0.015** | - |

Abbreviations: ASEs – anomalous self-experiences, DAT1 – dopamine transporter, TLEs(-) – negative history of traumatic life events, TLEs(+) – positive history of traumatic life events
